# Effects of Overweight/Obesity on Motor Performance in Children: A Systematic Review

**DOI:** 10.3389/fendo.2021.759165

**Published:** 2022-01-20

**Authors:** Waleska Maria Almeida Barros, Karollainy Gomes da Silva, Roberta Karlize Pereira Silva, Ana Patrícia da Silva Souza, Ana Beatriz Januário da Silva, Mariluce Rodrigues Marques Silva, Matheus Santos de Sousa Fernandes, Sandra Lopes de Souza, Viviane de Oliveira Nogueira Souza

**Affiliations:** ^1^ Programa de Pós-graduação em Neuropsiquiatria e Ciências do Comportamento, Universidade Federal de Pernambuco (UFPE), Recife, Brazil; ^2^ Centro Integrado de Tecnologias em Neurociência (CITENC)–Centro Integrado de Tecnologia e Pesquisa (CINTEP)–Centro Universitário Osman Lins (UNIFACOL), Vitória de Santo Antão, Brazil; ^3^ Programa de Pós-graduação em Nutrição, Atividade Física e Plasticidade Fenotípica, Universidade Federal de Pernambuco (CAV) - UFPE, Vitória de Santo Antão, Brazil; ^4^ Núcleo de Nutrição, Centro Acadêmico de Vitória, Universidade Federal de Pernambuco (CAV) - UFPE, Vitória de Santo Antão, Brazil

**Keywords:** obesity, metabolic syndrome, pediatric obesity, body mass index, motor skills

## Abstract

Childhood obesity is a serious public health problem. Childhood obesity and overweight are associated with the appearance of coordination deficit disorder and can cause impaired motor performance. We searched online databases for all related articles using comprehensive international databases from the Medline PubMed Institute, Web of Science, ScienceDirect, SCOPUS, and PsycINFO up to December 20, 2020. Overall, 33 studies were included in this systematic review. The present review demonstrated that children with higher percentage of body fat had lower levels of moderate to vigorous physical activity, as well as decreased levels of gross motor coordination, as shown by tests for neuromuscular performance. These results corroborate the hypothesis that overweight and obesity in children and adolescents are associated, not only with insufficient performance during gross motor coordination activities, but also with a greater risk to physical health.

**Systematic Review Registration:**

[https://www.crd.york.ac.uk/prospero/], identifier [CRD42020182935].

## Introduction

Childhood overweight and obesity are one of the greatest public health challenges worldwide. The World Health Organization estimates that approximately 70 million children will be overweight or obese by 2025, as children below 5 years old have shown a rapid increase in the development of overweight and obesity in recent years (1). Childhood is a critical period for the development of overweight and obesity. Increased consumption of unhealthy sugar, sodium, and fats, in addition to ultra-processed foods, including sugar-sweetened beverages and high-energy, nutrient-poor packaged foods have been strongly associated with weight gain and several nutrition-related non-communicable diseases (2). The high rate of obesity is associated with an increase in the development of some disease conditions such as systemic arterial hypertension (3), insulin resistance (4), and stroke (5). In addition to these conditions, obesity can affect physical parameters such as motor performance and gross motor coordination, as they seem to be directly related to regular physical activity and body composition in children and adolescents (6).

Motor coordination corresponds to the congruous interactions between the nervous, skeletal, and sensory muscle systems, in order to produce precise motor actions, in addition to quick reactions to everyday situations, which involves proper development of muscle strength and the proper selection of muscles that control the performance of the movement (7). Notably, motor performance in childhood and adolescence may be related to the programming of physiological systems in adult life (8, 9).

Motor competence, on the other hand, is the ability to perform different motor actions, including coordination and gross motor skills (10). Gross motor competence is often defined as proficiency in a range of fundamental movement skills such as throwing, catching, and running, which are normally learned during preschool and early school years (11, 12). These provide a basis for children to develop more in specialized movement sequences, such those required in sports activities (13).

A growing body of studies have investigated the possible relationship between gross motor coordination and the level of adherence to participation in physical activity during adolescence. Most studies found a positive association between better performance in gross motor coordination and participation in physical activities (14, 15).

It is possible that children and adolescents with poor gross motor skills may not want to participate in physical activity, because it can be more challenging for. It is also plausible that among children with poor gross motor skills, sedentary activities (i.e., watching TV and computing games) may be more enjoyable options.

The muscle is characterized by plasticity and, therefore, is more likely to change its structure and function. In animals, accumulation of intramuscular fat caused stiffness in the muscle tissue, which caused less contractility and decreased strength in the gastrocnemius muscle (16). In humans, a longitudinal study carried out on growth and physical fitness related to health and motor competence in elementary school children showed that the pathways for the development of physical and motor fitness are related to the children’s body weight. Children who had a low or medium rate of development of physical fitness and motor competence were more likely to develop overweight or obesity at the end of primary school, regardless of sex and body mass index at baseline (17).

In this context, it is necessary to clarify how environmental factors can influence the appearance of overweight and obesity; in addition, it is necessary to understand the relationship between overweight and obesity and motor performance in childhood (18–20). Core motor tasks include bilateral and upper limb coordination, strength, balance, speed, and running agility.

Motor skills are acquired from the physiological maturation of the neuromuscular system and environmental factors (21) and correspond to a group of coordinated movements that children begin to learn during early childhood and involve locomotor skills and object control. Locomotor skills are used to move the body through space, such as running, galloping, and jumping. The object control task is the ability to manipulate and project objects such as throwing, catching, dribbling, kicking, hitting, and rolling (22).

Although the genetic and biological determinants of obesity can interact throughout life, the process that regulates the developmental trajectories of other potentially important behavioral factors linked to the status of body weight has not been investigated.

Another aspect to be noted is that few studies have explored the contribution of current body composition to motor performance of the research participants. Understanding the relationship between overweight and obesity and children’s physical activity can guide the development of interventions at different levels that may provide a better chance of increasing the levels of physical activity in the population. Therefore, the objectives of this study were to analyze the influence of overweight and/or obesity on motor performance and gross motor coordination in children and adolescents.

## Methods and Materials

The protocol for this systematic review been published online (https://www.crd.york.ac.uk/prospero/) in PROSPERO (registration number CRD42020182935) and was reported as per Preferred Reporting Items for Systematic Reviews and Meta-analysis (PRISMA) (12).

### Search Strategy

This review was conducted in two phases, which included selection of studies followed by data extraction. Studies were selected from the search in the electronic databases Medline/PubMed (National Library of Medicine/Analysis of Medical Literature and Online Recovery System), Web of Science, ScienceDirect, SCOPUS, and PsycINFO, which was carried out in December 20, 2020. The following MeSH terms in Medline, PubMed, and DeCS in other databases were used as search filters: “obesity”; “pediatric obesity”; “metabolic syndrome”; “nutritional and metabolic diseases”; “body mass index”; and “motor skills”.

### Selection of Studies

Selection of studies was performed independently by WB and RS, according to the following inclusion criteria: (a) original articles addressing metabolic changes related to motor skills; (b) studies assessing individuals aged between 5 years old and 14 years and 11 months old; (c) studies with control and experimental groups (overweight and/or obesity); and (d) articles with a sample size of less than 30 individuals. No language or period of publication was set. However, a search filter was activated for viewing studies performed only in humans. The following PICOS criteria were established: Population: children and adolescents; Intervention/exposure: motor training; Comparison: between sexes; Results: overweight/obesity, motor coordination; Study design: cross-sectional and longitudinal studies. Initially, the studies were pre-selected according to titles and abstracts. In the next stage of the study selection phase and after excluding duplicate articles, texts considered eligible were read in their entirety.

Data were collected from the selected studies based on the characteristics of the studies, the results, and the components used to assess the intervening factors were verified. For the qualitative synthesis of the data, the following characteristics of the studies were used: author’s name, year of publication, country, age variation, sex, nutritional status, total population, analyzed variables, body composition, and motor performance results.

### Data Extraction

Selected abstracts were submitted to the second stage of analysis, in which two independent researchers reviewed the articles completely and, by consensus, excluded articles that did not meet the criteria. The following data from eligible articles were extracted: characteristics of the sample (mean age, distribution between sexes, and nutritional status), materials and methods (analyzed variables), and the main results found related to body composition and motor performance. The data extracted from the articles were collected using a standardized method among the authors. It was not possible to perform a meta-analysis in the present study, since there was substantial sample heterogeneity, in addition to the variability in the age range of the population of the studies, which could hinder the reliability of a meta-analysis.

### Risk of Bias

The risk of bias was established through of a critical analysis of the studies selected using seven criteria for a methodological judgment supplied by the software Revman 5.3.0 program the Cochrane Handbook 23, developed for systematic reviews and available for free download (https://training.cochrane.org/online-learning/core-software-cochrane-reviews/revman/revman-5-download). Among the criteria that structure the bias assessment are (1) random sequence generation, (2) allocation concealment, (3) blinding of participants and personnel, (4) blinding of outcome assessment, (5) incomplete outcome data, (6) selective reporting, and (7) other bias.

## Results

### Study Selection

**Figure 1:**
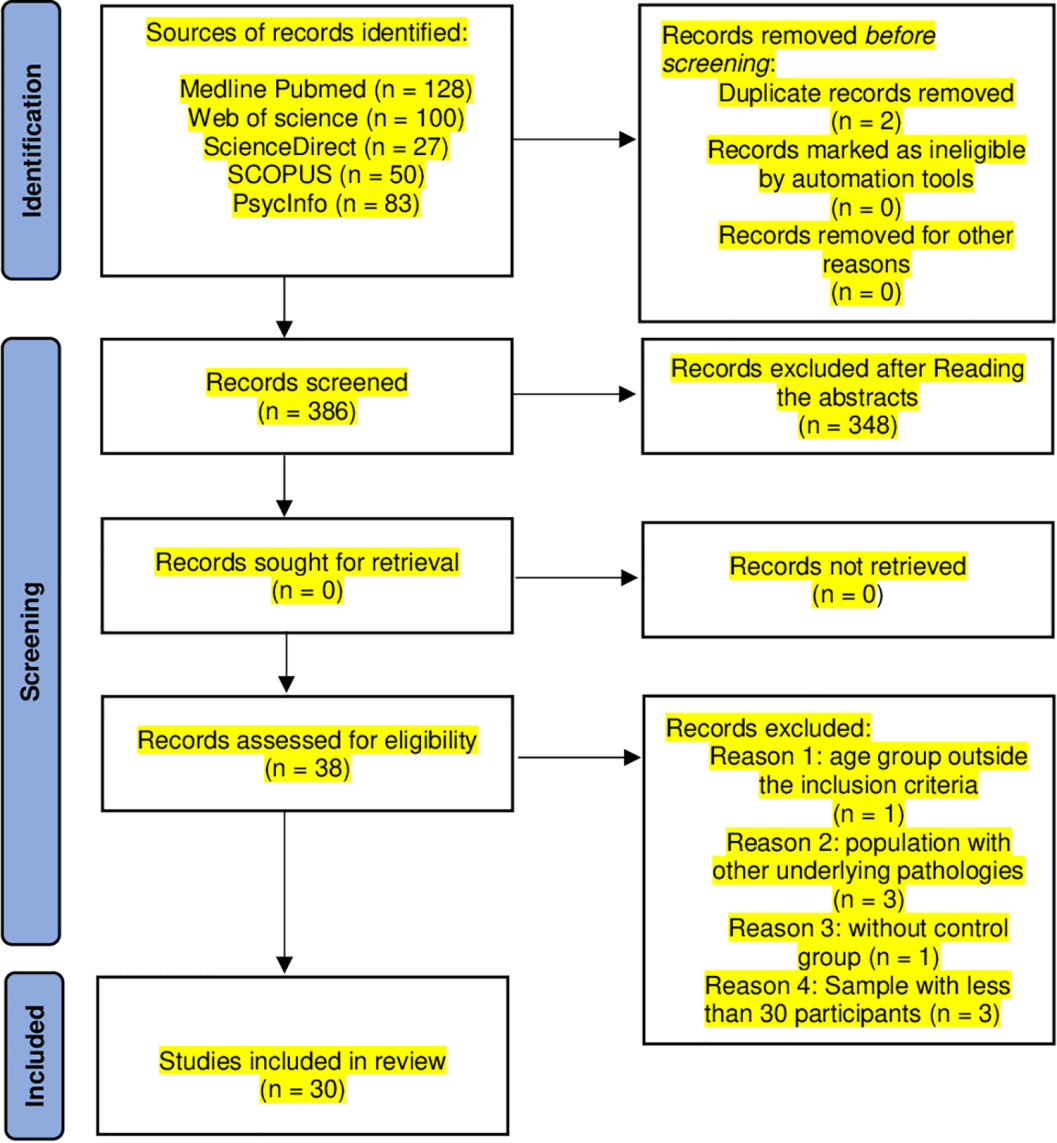
A flow diagram of the selection strategy.

A total of 388 studies were identified in the literature search. Two duplicates were found. Of these 386 studies, 38 met the inclusion criteria based on the title and abstract. Finally, 33 studies (**Figure 1**) were included in this review.

### Description of Included Studies

Among the main findings of this review, 1 of the 30 selected articles included children aged between 5 and 7 years old (23), 15 assessed children with ages between 7 and 14 years old (20, 24–40), 6 articles included children between 6 and 10 years old (41–46), and 5 included children in other age groups (47–51). All studies were conducted with children of both sexes (20, 23–50, 52–55) (**Table 1**).

**Table 1:** Descriptions of the studies included in the systematic review: age, sex, and nutritional status.

Author, year	Age range	Sex		Nutritional status			
		Female	Male	Malnutrition	Eutrophic	Overweight	Obesity
(23)	5 years	324	370	59 (23 girls) moderate: 41 (22 girls)	410 (200 girls)	92 (45 girls)	60 (17 girls)
(41)	6.5–7.2 years	369	412	8.1% (*n* = 63)	75.9% (*n* = 593)	8.8% (*n* = 69)	7.2% (*n* = 56)
(42)	6–10 years	69	86	–	–	27.1% girls	12.9% girls
						16.3% boys	14.0% boys
(47)	Data performed at two different ages: 5 and 10 years	307	361	–	–	5 years: 20.4%	5 years: 21.7%
						10 years: 22.9%	10years: 18.1%
(48)	6–14 years	2,787	2,351	–	–	Subjects with high BMI: 1,526	
(43)	6–8 years	204	200	–	–	14.7% girls	
						11% boys	
(54)	7–11 years	155	178	–	205	72	54
(24)	8–11 years (Three years of intervention)	108 (3rd year)	123 (3rd year)	–	–	3rd year: 23.4% girls and 23.5% boys	3rd year:10.6% girls and 13.9% boys
		108 (5th year)	126 (5th year)			5th year: 21.4% girls and 28.9% boys	5th year: 4.1% girls 3.9% boys
(49)	6–14 years	657 (51.5%)	619 (48.5%)	–	–	20.70% girls	5.02% girls
						17.69% boys	7.47% boys
(26)	9–12 years	Typical development:456	631	–	70.0%	22.4%	7.5%
		Disorders of motor coordination and balance: 93	85	–	61.8%	23.0%	15.2%
		Disorders of motor coordination and balance: 186	143	–	66.6%	24.3%	9.1%
(27)	5–13 years (1st evaluation)	1st evaluation: 1.188	1.329	Longitudinal study: significant inverse associations within the follow-up subsample participants between *z* scores of BMI and KTK MQ at each point in time (i.e., baseline and follow-up) as well as over the 2-year course			
	7–13 years (2nd evaluation)	2nd evaluation: 371	383				
(20)	10 and 14 years (accompaniment)	318	348	–	10 years: 507	116	43
					14 years: 486	126	54
(28)	10–13 years	107	132	–	132 (56 girls)	–	107 (51 girls)
(29)	9–13 years	268	322	Children with coordination disorder: ↑			
				BMI scores			
(44)	6–10 years	48%	52%	–	50	42	8
(30)	7–10 years	89	64	–	–	35	118 (65 girls)
(31)	9–10 years	951	1078	–	1,154 (577 girls)	434 (230 girls)	441 (144 girls)
(32)	9–11 years	1st wave: 1,120	1,158	–	–	30.1%	9.7%
		2nd wave: 1,094	1,133			31.2%	11.0%
		3rd wave: 1,094	1,133			29.6%	10.0%
		4th wave: 1,032	1,054			32.1%	10.5%
		5th wave: 1,032	1,059			32.3%	9.8%
(33)	11– 14 years	120	140	–	103 (49 girls)	86 (40 girls)	71 (31 girls)
(34)	8–10 years	105	105	–	105 (52 girls)	105 (53 girls)	–
(45)	Started: 6.8 ± 0,4 years	301	314	7.5%	77.8%	8.1%	6.6%
(50)	5–12.8 years	268	272	–	273	202	65
(53)	9–14 years	268	322	–	–	90 (overweight/obese)	
(46)	6.70 ± 0,42 years	278	280	8.1%	78.1%	8.1%	5.7%
(52)	6–11 years	335	341	04.28% (*n* = 29)	68.77% (*n* = 465)	11.24% (*n* = 76)	12.28% (*n* = 83)
(36)	10.4 ± 0.6 years	42.7%	57.3%	–	177	36 (overweight/obese)	
(55)	9–12 years	281	315	BMI only high than> 19.9			
(38)	7–10 years	343	313	Children who eat breakfast almost every day have better functional motor skills and a lower BMI than children who do not regularly eat breakfast			
(39)	7–10 years	198	182	Thinness: 4	325	35	Obesity: 10
				High thinness: 2			Severe obesity: 4
(40)	7–14 years	3,294	3,623	–	–	23.2% (overweight/obese)	

### Risk of Assessment

**Figure 2:**
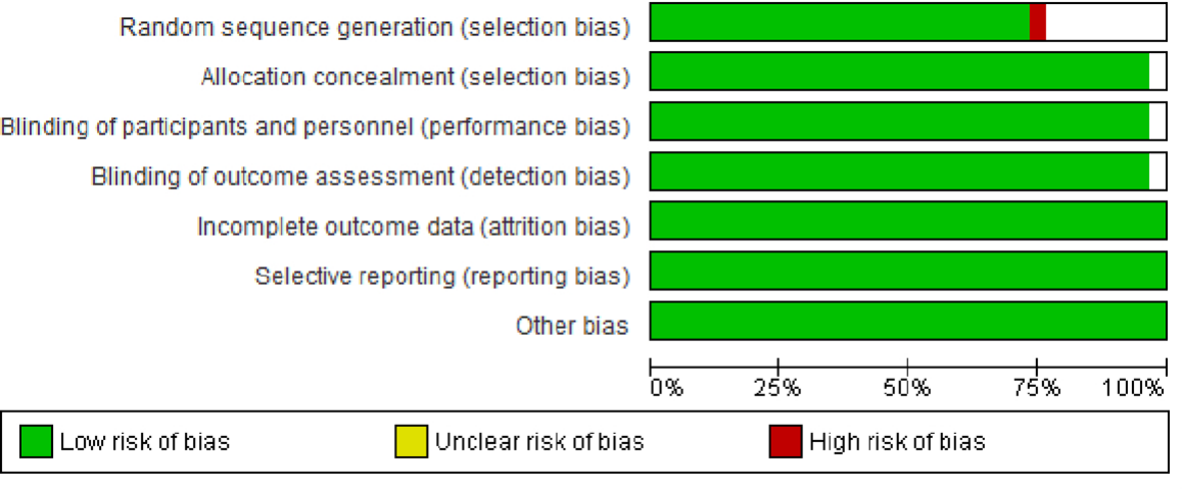
Risk of bias graph: review of authors’ judgements about each risk of bias item presented as percentages across all included studies.

**Figure 3:**
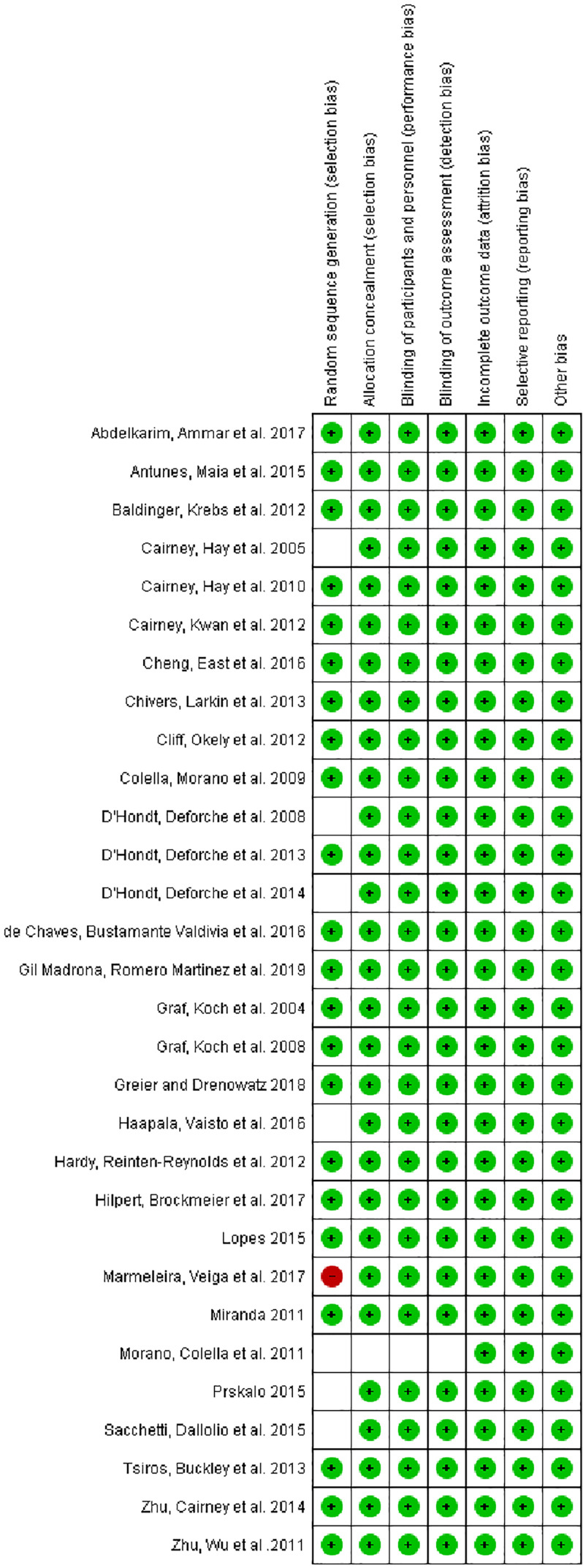
Risk of bias summary: review of authors’ judgements about each risk of bias item for each included study.

No studies with low risk of bias were excluded. The results are shown in **Figures 2** and **3**.

### Nutritional Status and Age Group

The classification of the relationship between nutritional status and age group was heterogeneous among the selected articles. An article that included children between 5 and 7 years old found that most participants had normal nutritional status (23). In children between 7 and 14 years old, two articles reported an inverse association between BMI and motor coordination (27). In another article, children who ate breakfast almost every day had better functional motor skills and a lower BMI than children who did not eat breakfast regularly (38). Overweight was more prevalent in three articles (20, 24, 32), overweight and obesity in three articles (33, 35, 40), normal and overweight in one article (34), normal and obesity in one article (37), and obesity in one article (30), and in most studies, participants were classified as having normal weight (25, 26, 28, 31, 36, 39). In children between 6 and 10 years old, our analysis revealed a higher prevalence of normal weight (41, 44–46), while in two studies, children were classified as overweight and obesity (42, 43). Other articles had a different age range from those already presented. A study of children 5 and 10 years old found that 21.7% of children had obesity at 5 years, and at 10 years old, 22.9% were overweight (47). Another study with children 5–12.8 years old found that the majority of the population studied was eutrophic (50), whereas in another, the majority had overweight and obesity (47); in one study, 1,526 out of 5,138 children evaluated had high BMI (48).

This systematic review investigated the characteristics of body composition and motor performance in children, without orthopedic or neurological changes, notably related to gross motor coordination with or without exposure to physical activity. The results of analysis, specifically the main characteristics of the included studies, were organized according to the correlation between body composition and motor performance (**Table 1**).

### Body Composition Related to the Motor Performance of Children and Adolescents

Based on the theory of developmental plasticity, overweight and/or obesity in children and adolescents can interfere with motor performance, alter postural control, and, consequently, modify the state of motor coordination of these individuals. Taking this into account, six included studies assessed the research participants’ motor performance using running speed and agility tests such as the six-minute running test, TUDS (timed ascent and descent test), and other explosion tests (28, 34, 35, 37, 43, 54). In one of these studies, the authors found a relationship between an increase in the percentage of body fat and a decrease in the levels of moderate to vigorous physical activity (43).

Another study observed a decrease in the levels of static strength and explosive power in girls 7–11 years old with obesity, as well as in boys 10–11 years old with obesity (54). Balance and muscle strength power represent important components related to the ability of physical fitness that have to be sufficiently developed throughout life to perform sports and daily activities to decrease the risk of injuries and falls (56). Furthermore, Tsiros et al. (28) found a decrease in motor performance in children with obesity during the TUG (timed up and go), 6MWT (six-minute walk test), and TUDS tests. Other studies found that using explosion motor performance tests, overweight children had worse performance in the long jump and 10- and 20-m sprints; in addition, individuals with an increased percentage of body fat showed lower indexes in the long jump and repetition during sit-ups, in addition to a deficit in perceived physical capacity (34, 35).

Prevalence of overweight and obesity associated with the levels of physical fitness among primary school age children in Assiut city CPA (Checklist of Psychomotor Activities), KTK (Body coordination test for children: Koërper Koordination Test für Kinder), MABC (Movement Assessment Battery Test for Children), and BOTMP-SF (Bruininks–Oseretsky Test of Motor Proficiency—Short Form) was investigated. Three studies used MABC to assess global motor coordination and balance (26, 31, 50) in a population of 540, 2,029, and 2,057 children, respectively. Another four found a greater propensity to develop deficit of coordination in children with greater accumulation of body fat, BMI, and obesity, successively (32, 42, 47, 53). However, most studies used KTK to assess gross motor coordination.

In this review, worse performance of gross motor coordination in children with obesity was observed (27, 41, 44–46, 48, 49, 55). One study investigated only the participants’ balance and found a decrease in balance skills with increasing body mass (52). Furthermore, overweight was negatively associated with lower overall performance of movements (24), while children with obesity had mild motor difficulties (20); overweight and obesity were related to less perceived and real physical competence (33), in addition to lower performance in side jumping, standing long jump, 20-m speed back-and-forth running (38), and decreased motor skills (40). Notably, a study including 380 children revealed that the association between nutritional status and motor classification in boys and girls was not significant, which, according to the authors, neutralizes any influence of nutritional status on motor classification (39) (**Table 2**).

**Table 2:** Descriptions of the studies included in the systematic review.

Author, Year	Country	Total population	Variables analyzed (Tests)	Results of body composition and motor performance
(23)	Spain	694 children	BMI (body weight and stature); CPA	GIRLS: Laterality: ↓ Childhood with malnutrition; dynamic coordination: ↓ Obesity Childhood; ↑ BMI worse results in motor execution; tonic postural control: ↓ Childhood with overweight/obesity; balance: ↑ Childhood with low weight; ↑ BMI ↓ respiratory control; visual-motor coordination between normal weight and Obesity/overweight childhood: ↑ Childhood with normal weight; spatial orientation: ↑ Childhood with normal weight *versus* overweight. BOYS: ↑ BMI ↑ Laterality; respiratory control ↓ and visual-motor coordination ↑ in Obesity/overweight childhood.
(41)	Germany	997 children	Height, body weight, BMI, SES, migratory background, motor skills: KTK and 6-min run, questionnaire on levels of daily and leisure activity, determination of sedentary activities	SES group ↓: ↑% obesity childhood, followed by the medium SES group and the high; obesity childhood group: ↑ migration history; overweight and obesity: ↓ gross motor development and resilience; how much ↑ SES, ↑ gross motor development; ↑ socioeconomic level, ↓ BMI and boys ↑ engine testing performance↑ computer/video game time: ↑ probability highest level of sports activity; history of migration: ↓ probability of participating in organized sports or being physically active at leisure
(42)	Portugal	156 children	%GM e IMC (dobras cutâneas, peso e altura); teste Bruininks-Oseretsky de Proficiência Motora - Forma Curta	↑ Cardiovascular disease risk: 27.5% girls and 24.4% boys excess body weight: 40% girls and 30.3% boys obesity childhood: ↓ gross motor skills and general motor proficiency;
(47)	Chile	668 children	BMI (height and weight); Motor skills: BOTMP-SF test	5 years: 20.4% overweight and 21.7% obesity; 10 years: 22.9% overweight and 18.1% obesity. Boys: ↑ total motor scores. Obesity childhood: ↓ gross and total motor skills (5 and 10 years) 5 obesity childhood years: ↓ performance in fine motor precision task (drawing lines). Childhood with obesity: ↓ motor skills from 5 to 10 years; ↓ motor proficiency at 5 years did not predict obesity or ↑ BMI. Overweight at 5 years was not enough to produce ↓ motor skills from 5 to 10 years; ↓ motor skill was associated with being overweight at 5 years
(48)	Peru	5193 adolescents sea level, *n* = 1299 altitude, *n* = 1292 jungle, *n* = 2602	BMI (height and weight); gross motor coordination: KTK; Physical fitness: Four EUROFIT battery tests (static and explosive muscle strength, flexibility, and speed/agility), abdominal muscle resistance of the Fitness gram battery and cardiorespiratory resistance of the American Alliance for Health, Physical Education, Recreation and Dance test battery; peak growth speed	Height, weight, and all motor performance test: ↑ with age except for sitting and reaching the boys outperform the girls in all tests. Girls: have 5 times + chances of ↓ gross motor coordination ↑ gross motor deficit with ↑ age; more mature girls and children: ↓ prone coordination deficits; ↑ BMI: ↑ prone to gross motor deficit. Children living at sea level or altitude: ↑ prone to gross motor deficit↑ flexible and ↑ strength: ↓ the probability of being diagnosed with deficit of gross motor coordination.
(43)	Finland	512 children	Fat body mass,% body fat, and lean mass; weight and height; physical activity: heart rate and movement sensor, PANIC Physical Activity questionnaire; 50-m shuttle test: running speed and agility; 15-m running test; Martin vigorimeter: handgrip strength; test of standing distance jump: explosive strength of the lower limbs; abdominal test; modified flamingo balance test; box and block test: manual dexterity and speed of movement of the upper limb; sit and reach test: flexibility of the lumbar and hamstring muscles; pubertal status.	Boys: more active, ↓ fat mass and% body fat, ↓ 50-m run time and 15-m run test time, ↑ absolute handgrip power, ↑ jump test standing jump, ↑ test errors balance of the modified flamingo, ↓ cubes moved in the box and block test and ↓ distance achieved in the sit and reach test. Children ↑% body fat and levels ↓ moderate to vigorous physical activity: ↓ neuromuscular performance running and jumping tests. Children ↑ body fat content and ↑ MVPA levels: surpass overweight and ↓ children active in the 15-m sprint and the long jump test. Children ↑% of body fat and levels ↓ of physical activity: ↓ neuromuscular performance
(54)	Croatia	333 children	Motor skills: polygon back - coordination, forward bending on a bench - flexibility, 15 ‘‘ manual touch - simple movement speed, long jump - explosive leg strength, flexed and static arm strength, abdominals - repetitive strength and high jump - MMII explosive force; % body fat (sum of subscapular skinfolds and triceps); body fat and fat-free mass; BMI (weight and height).	Obesity girls: between 9% and 13%. Obesity boys: range from 17% with age.7–9 years and 23% 10–11 years. Boys 7–9 years: N/S in motor skills when classified according to body weight. Boys 10–11 years old eutrophic: ↑ coordination, static, explosive and repetitive force. Girls 7–9 years old eutrophic: ↑ static strength and explosive power. Girls 10–11 years old eutrophic: ↑ static strength, explosive power, and coordination
(24)	Italy	231 children	Anthropometric measurements (height, weight, BMI) and motor skills: Sit & Reach test, Forward Roll Test, Forward Throw 2 kg Medicine-ball test, long jump test, 20-m running speed test.	Beginning of the study: 35.8% of children ↑ weight (23.4% overweight childhood; 12.4% obesity childhood); after intervention: ↓ to 29.3% (25.3% overweight childhood; 4% obesity childhood). N/S in the various motor skills. There was an association between BMI and flexibility of the hips and lower back (Sit & Reach Test) or total dynamic body coordination (Advance Test). Overweight childhood: ↑ segmental movements (positive association with BMI), ↓ overall movement performance.
(49)	Portugal	1,276 children	Gross motor coordination (MC): KTK; anthropometry: height and body mass; physical activity: Baecke’s questionnaire; and socioeconomic status (SES)	Overweight and obesity: 17.69% and 7.47%, respectively, for boys, and 20.70% and 5.02% for girls. Eutrophic children: overcome childhood with obesity in all tests of gross motor coordination. Gross scores when walking backwards and moving sideways: ↑ with age and performance boys ↑ when moving sideways
(26)	Taiwan	2,057 children	MABC test; anthropometry: height, body weight, waist and hip circumference, BMI	Manual dexterity and ball skills in girls: scores ↑ mastery of manual dexterity; most anthropometric data (weight, waist circumference): ↑ group with developmental coordination disorder and balance deficits; children in the group with developmental coordination disorder and balance deficits: 2× ↑ probability of being obese
(27)	Belgium	2,517 children initially *n* = 754 in the second evaluation	BMI, gross motor coordination: KTK, total physical activity: questionnaire	Performance ↓ in KTK at baseline predicted ↑ BMI *z* score; ↑ baseline BMI *z* score predicted ↓ KTK performance
(20)	Australia	666 children and adolescents Evaluated at 10 and 14 years old	Anthropometric measurements: height, weight, BMI; engine performance: MAND	14 years old eutrophic children group: ↑ general motor performance scores. 14 years: ↑ prevalence obesity childhood with mild motor difficulties. ↓ motor performance and BMI ratio; tasks + affected by BMI: those that involved a change in the center of mass; morphological restrictions of overweight and obesity affect the performance of motor tasks in tasks involving changes in the center of mass, but not static measures of strength
(28)	Australia	239 children Obese *n* = 107 Normal weight *n* = 132	Anthropometry: height, weight, BMI; body composition: dual-energy absorptiometry by x-rays; physical activity: uniaxial accelerometers; demographic/background information; activity capacity restrictions: TUDS; 6MWT, TUG; limitation of participation (performance): Multimedia Activity Recall for Children and Adolescents, QVRS	Obesity childhood: ↓ average accelerometry count, maternal education, and family income. Obesity childhood: ↑ mass, BMI, % fat and fat-free mass; obese group: restrictions on the ability to perform the TUG, the 6MWT and the TUDS; Obesity childhood: ↓ time in self-care activities and without physical difficulty in daily activities; obese: impaired quality of life
(29)	Canada	590 children and adolescents	Height, weight (BMI), and % body fat by bioelectrical impedance analysis; BOTMP-SF; active game participation: participation questionnaire	Youth with Developmental Coordination Disorder: ↑% body fat. Boys with Developmental Coordination Disorder: ↑ BMI of all young people. Boys with Developmental Coordination Disorder: ↑ active play participation associated with ↑ BMI and% body fatBoys with Developmental Coordination Disorder: opposite relationship is observed
(44)	Belgium	108 children	Anthropometry: body height, body weight, BMI, % body fat; level of gross motor coordination: KTK; FPAQ	Progression level of gross motor coordination over a period of 2 years was different, depending on the children’s weight status; eutrophic childhood group: ↑ progress; in addition to BMI (negative predictor), participation in sports organized within a sports club (positive predictor) determines the gross performance of motor coordination 2 years later
(30)	Australia	175 children	Anthropometry: height, weight, BMI; fundamental movement skills: TGMD-2 age groups: 7–8 years and 9–10 years; all other SFM: 6 to 7 years and 8 to 10 years	77% obesity childhood; boys: ↑ BMI and performance in object control skills; girls: ↑ proficiency in locomotor skills; all 12 skills in all age groups: domain prevalence was ↓ among overweight/Obesity childhood
(31)	Taiwan	2,029 children	Height, weight, % body fat; coordination: MABC	Boys and girls with obesity: ↓ general motor coordination, mainly in static and dynamic balance; boys: ↑ developmental coordination disorder (DCD) in the obesity group
(32)	Canada	2,278 children 1,979 performed the motor tests	Height, weight, BMI, waist circumference; identification of developmental coordination disorder: BOTMP-SF	Balance and total impairment score: ↑ obesity and overweight; girls: ↑ balance impairment score in obesity and overweight groups
(33)	Italy	260 children	Anthropometry: height, weight, BMI; self-physical description questionnaire: perceived coordination, body fat and sports competence; drawings of Collins Children’s Figures: body image; Perceived Physical Capacity Scale: strength, speed and agility and tests involving standing long jump, 2 kg medicinal ball toss, 10 × 5 m shuttle race and 20- and 30-m sprints.	Overweight and obesity girls: ↓ perceived and real physical competence, ↑ perceived body fat and ↑ body dissatisfaction eutrophic children: ↑ standing long jump performance, 20-m shuttle run and 30-m run. Obesity childhood: ↑ pitch performance
(34)	Italy	210 children Normal weight *n* = 105 Overweight *n* = 105	Height, weight, BMI; motor performance tests: 3 explosion tests (standing long jump, medicine ball throw, basketball throw; 2 speed tests: 10- and 20-m sprint; body image: children’s drawings of Collins; scale of perception of physical ability for children	Scale of perception of physical ability for children: overweight childhood showed ↑ average body discrepancy; overweight childhood explosion tests: ↑ ball and basketball performance; long jump and 10- and 20-m sprint: eutrophic childhood ↑ performance
(45)	Germany	615 children	Antropometria: altura, peso e IMC; testes motores: TC6; coordenação motora: KTK	Intervention schools: Overweight and Obesity childhood: ↓ motor test results on all tasks
(50)	Belgium	540 children	Anthropometry: height, weight, and BMI; fine motor control: MABC in two postural conditions different: sitting and standing in a tandem position on a balance beam (BB)	Tandem position on balance beam: ↓ obese score in seated condition: N/S between overweight and eutrophic scores performance in placing obese pins: ↓ when seated.
(53)	Canada	578 children	BOTMP-SF, % body fat, weight, height, and BMI	Children with coordination and balance deficit disorder: + prone to being overweight and obesity childhood (analyzing% body fat)
(46)	Germany	668 children	Anthropometry; gross motor development: KTK; resistance: TC6; children’s leisure assessment questionnaires	Boys with coordination and balance deficit disorder: risk factor for overweight and obesity in childhood and early adolescence
(52)	Egypt	676 children	Anthropometry: body height, body weight and BMI; physical fitness: DMT 6-18	Overweight childhood: 11.24%; obese: 12.28% running and strength skills: negatively + affected by ↓ body weight balance skills affected by ↑ body mass; weight and endurance skills: affected by abnormal ↑ or ↓ body weight
(36)	Áustria	213 classmates	Height, weight, and BMI; DMT 6–18: resistance, power, speed, coordination, and agility; 6MWT questionnaire participation in sports and use of media; migration status	Eutrophic childhood: 83% adolescents overweight/obesity participants: ↓ motor skills development Participants who lost weight or maintained normal weight: ↑ overall motor skill score over 4 years of follow-up + time using media eutrophic adolescents: ↑ performance in various tests of motor skills motor skills during the 4-year observation period: ↑ absolute performance more pronounced in eutrophic adolescents at baseline
(55)	Portugal	596 children	Anthropometry: weight, % fat, height, waist circumference and BMI; motor coordination: KTK; 20-m shuttle-run test: assess cardiorespiratory fitness	Girls: ↓ CM and ↑% body fat BMI, waist circumference, % body fat and waist/height ratio: related to ↓ CM in both sexes, except for the waist/height ratio after adjustments for girls
(38)	Switzerland	656 children	Coordinating and conditional skills: lateral jump, touch, standing jump, 20 m and shuttle run; weight, height, and BMI; nutritional research	Eutrophic childhood: ↑ running performance, side jump, long jump, and shuttle run. Low weight group: ↑ shuttle race performance. Obesity and overweight group: ↓ performance on 4 items of the motor functional tests (lateral jump, standing long jump, 20 m speed and shuttle run)
(39)	Brazil	380 children	Motor performance: MABC-2—manual dexterity, throw receive skills and static and dynamic balance skills. Antrhropometry: weight, height, and BMI	Male: ↑ movement difficulty. Between ages: association N/S; age ranges by skill compared: significant difference between age range and static and dynamic balance skills (between ages 7 and 9 and between ages 7 and 10) motor classification and nutritional status by sex: N/S, which neutralizes any influence of nutritional status on motor classification
(40)	Australia	6917 children	Demographic information: socioeconomic status (SES); fundamental movement skills: sprinting, vertical jumping side canter and jumping and object control skills (catching, throwing by the arm and kicking); cardiorespiratory endurance fitness: 20-m shuttle race test, parents reports of physical activities organized or not; validated physical activity recovery for adolescents questionnaire	Girls: ↑ low competence skills object control association with functional movement screen and inadequate cardiorespiratory fitness. There was no association between low competence and object control skills and overweight students/obesity. Motor skills: ↑ low overweight. Competence association/obesity; consistent associations for most individual motor skills

BMI, body mass index; CPA, Checklist of Psychomotor Activities; SES, socioeconomic status; KTK, Body coordination test for children, Koërper Koordination Test für Kinder); N/S, not significant; BOTMP-SF, Bruininks–Oseretsky Test of Motor Proficiency—Short Form; MABC, Movement Assessment Battery Test for Children; MAND, McCarron Assessment of Neuromuscular Development; TUDS, timed ascent and descent test; 6MWT, six-minute walk test; TUG, timed up and go; HRQoL, related quality of life; FPAQ, Flemish Physical Activity Questionnaire; TGMD-2, gross motor development test 2; DMT 6–18, German engine test/Deutscher Motorik Test; MLG, Fat-free mass; SLJ, standing long jump; MVPA, moderate to vigorous physical activity; MT, Hand movement time.

## Discussion

Overall, the results of this review confirmed the hypothesis that overweight and obesity can negatively affect motor performance and gross motor coordination in children and adolescents, although age, nutritional status, and the measures of motor performance analyzed were different among the investigated studies.

It is well recognized that motor performance in some tests is negatively affected by higher body weight (23, 53). In analyzing the magnitude of the relationships between gross motor coordination, physical activity, and physical conditioning, weight was strongly associated with age and sex in gross motor coordination tests (57, 58). A meta-analysis showed that age was positively associated with locomotion, object control, and stability skills. It is not surprising that the older children are, the better their skills, as long as they continue to participate in activities that develop these skills. Motor development in young children is influenced by biological maturation, and after this period, it depends more on practice and opportunity. Thus, it is conceivable that the relationship between age and gross motor competence may change over the developmental periods of early childhood, preschool, childhood, and adolescence. Notably, although primary evidence confirms age as a positive correlate in most aspects of motor competence, some studies (across all types of motor competence) have not found this relationship (59). One study that found age to be a negative correlate involved adolescents and suggested that the decline in girls’ motor competence was due to a reduction in the opportunity to be active (60). It then appears that gross motor coordination improves with age during middle childhood and adolescence, although there is a lack of consensus on sex-related differences between age groups and the gross motor coordination tests used.

In contrast to object control-related skills, which tend to be more static, locomotor activities involve changing or controlling a larger body mass that impedes functional movement and contributes to a higher rate of lower limb orthopedic changes, such as tibia rod and plantar pressure, among children with obesity (61). The negative association between gross motor activity and higher BMI may reflect the composition of assessments where the compound requires better motor coordination while moving and controlling the body, compared to object control skills. Sex, on the other hand, seems to relate differently to various aspects of gross motor competence. Male sex was considered a strong positive correlate of object control and motor coordination tasks, with pre-maturation biological differences being considered for boys and girls, especially in reference to skills such as throwing (62). Research has shown that, compared to girls, boys receive greater encouragement, support, and opportunities to engage in physical activity and sports at home and at school. Thus, girls’ opportunities to improve their gross motor skills may be limited (63, 64).

Biological and environmental factors can influence motor coordination, favoring both boys and girls. The activities performed by different sexes facilitate the performance in certain items of motor coordination; therefore, sex can be an intervening factor in motor performance. Regarding overweight and obesity, one of the hypotheses that can explain the interference in the performance related to gross motor coordination tasks is that during the tasks of supporting the body weight, there is a higher proportion of fat mass that must be supported or moved against the action of the force of gravity (65).

Another factor that can interfere with the performance in motor coordination is time, as can be seen in a longitudinal study that investigated the relationship between children’s weight and the level of gross motor coordination over time. Baseline measurements were collected from 2,517 children (5 to 13 years old, 52.8% boys). Measurements included the following: height and body weight for the calculation of BMI and gross motor coordination through KTK. After 2 years, 754 participants (7 to 13 years old, 50.8% boys) underwent anthropometric and KTK assessments again. There was a positive relationship between the worst motor performance at KTK at baseline and an increase in BMI. In addition, a higher baseline BMI score also predicted a decrease in KTK performance, suggesting that children’s weight negatively influences the level of gross motor coordination in the future and vice versa. Therefore, prevention and intervention initiatives through physical activity must consider this reciprocal causal relationship over the development time (50).

Furthermore, physical activity has a potential protective effect against the development of metabolic diseases during childhood and reduces the prevalence of cardiovascular diseases and diabetes, and morbidity and mortality of adult individuals prematurely (66). Thus, regular physical activity and adequate nutrition during the years of child growth and development increases the possibility of a healthy pattern of physical maturation consistent with a child’s genetic potential (67). Dudas et al. (2008) found that overweight children showed lower participation in sports clubs, while even more children with healthy weight were able to ride a bicycle.

In this perspective, this review demonstrated that children with a higher percentage of body fat had lower levels of moderate to vigorous physical activity, as shown by the neuromuscular performance in running and long jump tests (43). In addition, Tsiros et al. (28) performed a study on 239 children, of whom 107 had obesity and 132 had a healthy weight. They observed restrictions in the group with obesity regarding the ability to perform TUG, 6MWT, and TUDS. Morano et al. (33) evaluated 260 students between 11 and 14 years old through the questionnaire of physical self-description: perceived coordination, body fat, and sports competence; Collins Children’s Figures Drawings: body image; Perceived Physical Capacity Scale: strength, speed, and agility, and tests involving standing long jump, and 20- and 30-m sprints. Overweight and obese girls reported less perceived and real physical competence, a higher index of perceived body fat, and body dissatisfaction. Eutrophic childhood, on the other hand, showed better performance in standing long jump, shuttle run, and 20-m and 30-m run.

It is important to note that the mechanisms involving the neuroendocrine and musculoskeletal systems interact with each other and can explain the associations between weight and performance in gross motor coordination tests. Scientific literature demonstrates that stimuli from greater muscle activity are capable of promoting in their microenvironment the synthesis of chemical compounds called myokines. Among these, BDNF (brain-derived neurotrophic factor) and, recently, irisin stand out, because they are able to overcome the blood–brain barrier and can promote a positive outcome in both the cognitive and motor domains (68, 69).

For several years, muscles were considered targets for hormonal action; however, there is growing evidence that muscles, in a retrograde manner, exert unique forms of control over the CNS that affect motor behavior. Therefore, increasing evidence indicates that neural and muscular systems maintain some degree of plasticity throughout life, demonstrating that environmental factors influence the development of the musculoskeletal system and, as a consequence, motor performance.

## Conclusion

Our results corroborate the hypothesis that overweight and, especially, obesity in children and adolescents are associated not only with insufficient performance during gross motor coordination activities, but also with an increased risk to physical health. It is, therefore, necessary to prevent childhood obesity and reduce the weight of affected children, and promote healthy eating and physical activities in daycare centers, schools, and homes. To be effective, in addition to the educational sector, all sectors of society must be mobilized so that the negative effect of commercial food products on children’s diets will be reduced.

## Data Availability Statement

The original contributions presented in the study are included in the article/supplementary material. Further inquiries can be directed to the corresponding author.

## Author Contributions

WB and MSF contributed to research conception, data collection, interpretation of results, and critical review of the article. RS, KS, ASS, MS, and AS contributed to data analysis and interpretation, drafting, and critical review of the article. SS and VO contributed to data collection and critical review of the article. All authors contributed to the article and approved the submitted version.

## Funding

The authors thank the “Coordenação de Aperfeiçoamento de Pessoal de Nível Superior (CAPES) – Edital PROPG n° 02/2021” and the “Conselho Nacional de Desenvolvimento Científico e Tecnológico” (CNPq) for their financial support. We would like to thank Editage (www.editage.com) for English language editing.

## Conflict of Interest

The authors declare that the research was conducted in the absence of any commercial or financial relationships that could be construed as a potential conflict of interest.

## Publisher’s Note

All claims expressed in this article are solely those of the authors and do not necessarily represent those of their affiliated organizations, or those of the publisher, the editors and the reviewers. Any product that may be evaluated in this article, or claim that may be made by its manufacturer, is not guaranteed or endorsed by the publisher.
